# Successful Utilization of Levodopa in HIV-Induced Parkinsonism

**DOI:** 10.7759/cureus.11825

**Published:** 2020-12-01

**Authors:** Mohammad Almajali, Fawwaz Almajali, Jafar Kafaie, Pratap Chand

**Affiliations:** 1 Neurology, Saint Louis University School of Medicine, St. Louis, USA

**Keywords:** parkinson' s disease, hiv aids, altered mental state

## Abstract

We report a case of a 63-year-old African American female patient with a past medical history of treatment naïve human immunodeficiency virus (HIV). She was referred to our hospital with altered mental status and rigidity with a history of progressive ambulation difficulties and decreased verbal output over the previous months as reported by her son. Her clinical presentation and brain MRI were consistent with HIV encephalopathy with bilateral basal ganglia involvement and HIV-induced parkinsonism. We initiated a trial of carbidopa/levodopa along with highly active antiretroviral therapy (HAART) (emtricitabine-tenofovir and dolutegravir). In the following three weeks, she demonstrated dramatic improvement, both clinically and radiologically. She tolerated carbidopa/levodopa well with no behavioral or neurological side effects. This case illustrates the safe utilization of carbidopa/levodopa in treating parkinsonism in an adult female patient with HIV encephalopathy.

## Introduction

Neurological disorders are a common complication in human immunodeficiency virus (HIV) infection. It most commonly includes opportunistic central nervous system (CNS) infections and neoplasms. Additionally, a wide variety of movement disorders have been recognized, including tremors, parkinsonism, hemichorea-ballismus, myoclonus, and dystonia [1]. One retrospective study performed in a tertiary referral center reported a 3% incidence in movement disorders in HIV positive patients [1]. Parkinsonism was found to be the most common movement disorder to affect HIV-infected patients and occurs in 5% of cases [2]. The same study also found that parkinsonism occurred in severely immunosuppressed patients with a cluster of differentiation 4 thymus (CD4 T) cell count of fewer than 40 cells/mm3 [2].

Different etiologies were identified for HIV-induced parkinsonism. Some have speculated that exposure to neuroleptics and antiemetic medications may predispose them to drug-induced parkinsonism, as can intracerebral opportunistic infections, such as toxoplasma, Cryptococcus, and progressive multifocal leukoencephalopathy [3,4]. A few studies have reported isolated HIV-induced parkinsonism without exposure to opportunistic infections or to antidopaminergic medications as the basal ganglia are a vulnerable target to HIV [1,5].

The HIV-induced parkinsonism presentation is often different from idiopathic Parkinson’s disease in that it includes symmetrical bradykinesia and rigidity, early presentation of postural/gait instability, and lack of the typical pill-rolling rest tremor. The course of illness is variable and treatment should be focused on evaluation for potential underlying opportunistic infections and medication review for extrapyramidal side effects. 

The treatment of HIV-induced parkinsonism is controversial. Many studies consider the highly active antiretroviral drug regimens alone as the effective treatment for HIV-induced parkinsonism [5,6] while a few studies suggest that patients would benefit from levodopa concurrently with highly active antiretroviral therapy (HAART) [7,8].

Here, we present a case of isolated HIV-induced parkinsonism in an African American female patient who initially presented with altered mental status and weakness. The case in this report is unique in its complexity, masked presentation, and successful treatment with carbidopa-levodopa therapy concurrently with starting HARRT without significant adverse effects.

## Case presentation

Our case is of a 63-year-old African American female patient with a past medical history of HIV and diabetes type II (not on any treatment). She was diagnosed with HIV two years prior to this presentation, and she was not taking any HAART. She initially presented to an outside hospital (OSH) with altered mental status and generalized weakness that started 3-4 days before admission. Her son stated that she had developed progressive ambulation difficulties for the last few months and decreased verbal output with a possible history of fall, given that the patient was living alone.

At the OSH, she was lethargic and non-responsive. She was febrile (101.2) and was hence started on empirical antibiotics (vancomycin, cefepime, metronidazole, and acyclovir) preceded by lab workup. OSH results were significant for CD4 absolute: 45 cells/mcL, HIV ribonucleic acid (RNA) viral load: 4,390,000 copies/mL. Repeated after seven days: 1,570,000 copies/mL. Blood cultures showed 1/2 coagulase-negative Staph. Hepatitis panel (hepatitis A, B, and C antibodies as well as hepatitis B surface antigen), rapid plasma reagin, serum cytomegalovirus (CMV) were negative. Cerebral spinal fluid (CSF) analysis revealed 49 mg/dL glucose, 140 mg/dL protein, 0 red blood cells (RBC)/mcL, 0 white blood cells (WBC)/mcL, clear/colorless and an opening pressure of 12 cm H2O. CSF gram stain and culture, CSF cryptococcus antigen, John Cunningham (JC) virus polymerase chain reactions were negative as well. Stool studies were negative for Giardia and Cryptosporidium.

Computed tomography (CT) scan of the head showed no acute intracranial pathology. CT of the cervical spine showed no cervical spine fracture. MRI of the brain showed multiple punctate foci of restricted diffusion with associated edema in the bilateral basal ganglia with additional small areas of abnormal signal in the subcortical white matter that are nonspecific and confluent signal abnormality in the periventricular white matter. Routine electroencephalogram (EEG) showed abnormal EEG based on poly frequency rhythms and poor organization, in keeping with a state of encephalopathy.

The suspicion for an infectious process was high; she was kept on broad-spectrum antibiotics (vancomycin, cefepime, metronidazole, and acyclovir), for a total of five days until cultures came back negative but no significant clinical improvement was noted. She was transferred to our hospital for persistent encephalopathy of unclear etiology.

At our hospital, she was still encephalopathic, non-responsive, and not following commands. She was noted to have rigid neck and limb muscles with bradykinesia. She had normal deep tendon reflexes and no tremors were seen. Laboratory studies were significant for a hemoglobin of 8.4, WBC of 5.5, and HIV RNA viral load of 3080000 copies/mL. Blood bacterial cultures and mycobacterial cultures were negative. Serum CMV, human herpesvirus (HHV) 8, Toxoplasma, Blastomyces, Histoplasma antibodies, and rapid plasma reagin were negative as well. CSF analysis revealed 214 mg/dL protein, 67 mg/dL glucose, 1 WBC/mcL, 4 RBC/mcL and an opening pressure of 18 cm H2O. CSF herpes simplex virus (HSV) 1 and 2, varicella-zoster virus (VZV), Epstein-Barr virus (EBV), CMV, JC virus, and enterovirus polymerase chain reactions were negative. CSF toxoplasmosis antibodies, cryptococcus antigen, West Nile virus immunoglobulin (Ig)M, and IgG were negative as well. CSF cultures were negative, including bacterial, mycobacterial, and fungal.

**Figure 1:**
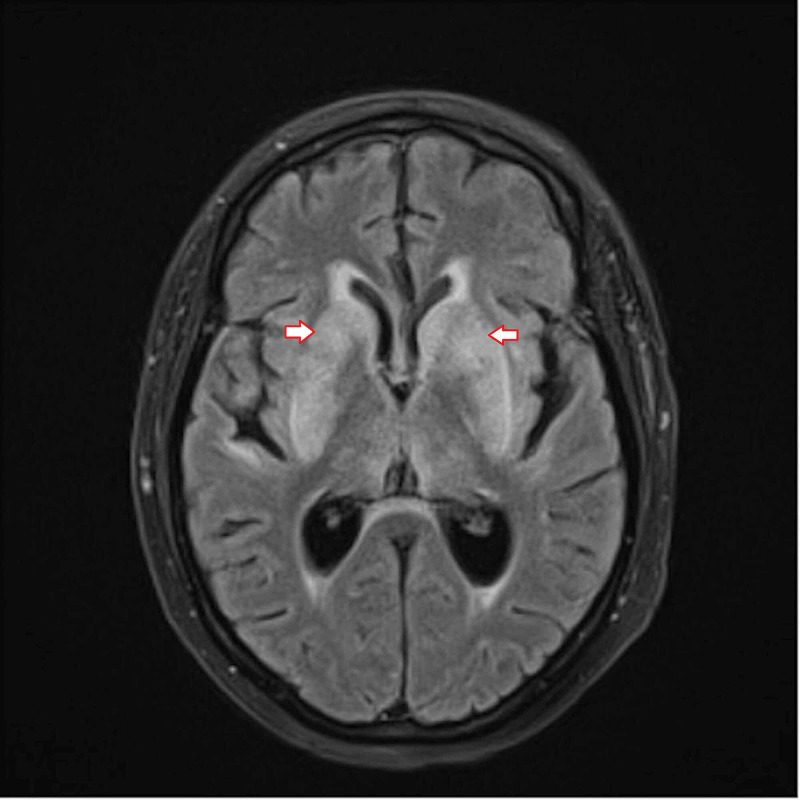
Brain MRI on initial presentation Grossly symmetric high T2 fluid-attenuated inversion recovery (FLAIR) signal abnormality involving bilateral basal ganglia (with greater involvement of caudate nuclei and putamen than globus pallidus internus), thalami, portion of corpus callosum, portion of medial temporal lobes, and perisylvian/insular cortex regions.

Brain MRI showed grossly symmetric high T2 fluid-attenuated inversion recovery (FLAIR) signal abnormality involving bilateral basal ganglia with greater involvement of caudate nuclei and putamen than globus pallidus internus (Figure 1).

The clinical picture of rigidity and bradykinesia with basal ganglia lesions were suggestive of HIV-induced parkinsonism, thus she was started on HAART (emtricitabine-tenofovir DF 200-300 mg tablet one tab once daily and dolutegravir 50 mg tablet one tab once daily) and carbidopa-levodopa 25/100 mg one tab TID.

**Figure 2:**
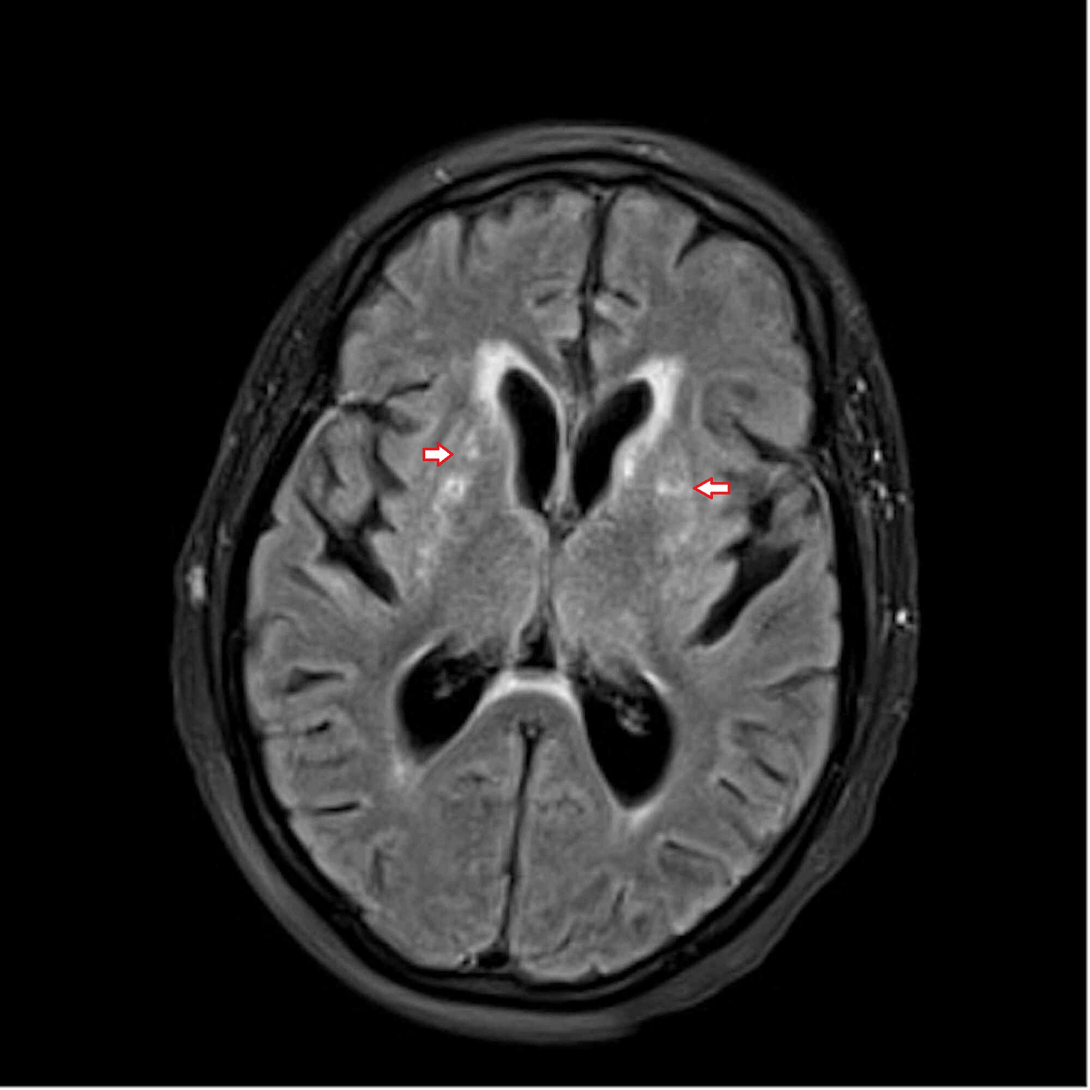
Brain MRI after 16 days Interval evolution of the symmetric signal abnormality and scattered foci of restricted diffusion involving the bilateral basal ganglia, the caudate nuclei and adjacent periventricular and insular regions with resolution of the edema and decrease in the T2/fluid-attenuated inversion recovery (FLAIR) signal intensity and resolution of the restricted diffusion.

She started to improve gradually; she regained consciousness and became more alert and interactive. Brain MRI after 16 days showed interval evolution of the symmetric signal abnormality of the bilateral basal ganglia (Figure 2).

After three weeks, she was fully alert and oriented and tolerating oral feeding. Her hypophonia, rigidity, and bradykinesia were improving. She was able to work with physical therapy with mild to moderate assistance and was discharged to a rehabilitation center. After two months on follow up, she was doing well and functioning independently. She stayed compliant with HAART and carbidopa-levodopa and showed dramatic improvement in bradykinesia and rigidity. She did not demonstrate any dyskinetic or psychiatric side effects. Routine EEG showed no epileptiform abnormalities or electrographic seizures.

## Discussion

Movement disorders in HIV patients have increasingly been recognized and include tremors, parkinsonism, hemichorea-ballismus, myoclonus, dystonia, and drug-induced movement disorders [3,4]. Mirsattari et al. found that parkinsonism occurred in 5% of HIV-infected patients, and it mainly occurred in severe immunosuppression with CD4 T cell count of fewer than 40 cells/mm3 at the time of diagnosis [2]. Mattos et al. observed that the number of HIV-related movement disorders has declined since 2000 due to the introduction of HAART [9].

Here, we present a case of HIV-induced parkinsonism in an African American female patient successfully treated with carbidopa-levodopa and concurrent HAART to prevent disease progression. Follow up after two months with continued carbidopa-levodopa and HAART revealed dramatic improvement. The patient had not received any dopamine blocking agents and there was no evidence of opportunistic infections. Therefore, her symptoms are likely due to direct HIV infiltration of the basal ganglia.

Isolated HIV-induced parkinsonism cases without the exposure to opportunistic infections or dopaminergic blocking medications have been reported, as the basal ganglia are a vulnerable target to HIV [1,5]. The neuropathology was linked to viral proteins, especially glycoprotein (gp)120 and transactivator of transcription (Tat) proteins, which are released from infected microglia/macrophages and astrocytes. Gp120 causes excitotoxicity and dopaminergic dysfunction by different mechanisms, including N-methyl-d-aspartate (NMDA) receptors hyperactivation and impairment of extracellular glutamate reuptake [10]. Tat proteins also affect the dopamine system by inhibiting the expression of tyrosine hydroxylase leading to alternation in the dopamine system and impairment of the dopamine transporters, thus reducing dopamine overflow and neurotoxicity by activation of the D1 mediated pathways [11]. HIV-infected microglia and macrophage, which are more prominent in the basal ganglia compared to other brain regions, played an important role by producing cytokines (tumor necrosis factor (TNF)-α, interleukin (IL) 6, IL-1β) that lead to oxidative stress, excitotoxicity, distress of cellular defense mechanisms, subsequent neuronal damage and apoptosis [12].

HIV-infected patients were found to have as much as 25% of nigral neuronal loss, even in asymptomatic patients [13]. They were also found to have a reduction of dopamine in the CSF without neurocognitive deficits [13].

A significant relationship has been established between HIV viral load and its neurological manifestations [14,15]. Optimal suppression of viral load in antiretroviral naïve HIV patients is achieved after at least three months of initiation of HAART [16]. Highlighting the long course of neurological improvement with HAART, several case reports demonstrated improvement of HIV-induced parkinsonism over a few months after initiation of HAART alone [6,17]. It is likely that the early improvements in motor and cognitive symptoms in our patient are most likely related to levodopa rather than the antiretroviral therapy.

To our knowledge, there are no reported case series on adults with HIV-induced parkinsonism treated with levodopa to draw any definite conclusions regarding its efficacy. However, two adult case reports [8,18] and a series of five pediatric patients with HIV-induced parkinsonism demonstrated good response to levodopa [7]. Treatment of HIV-induced parkinsonism has both psychiatric (psychosis, mania, worsening confusion, etc.) and dyskinetic adverse effects [17]. One study demonstrated that the combination of carbidopa-levodopa and protease inhibitors (indinavir) were associated with peak-dose dyskinesias due to the inhibitory effects of protease inhibitors on the cytochrome p450 system [18]. Some studies raised some concerns about the activation of HIV by levodopa, leading to increased viral load in simian immunodeficiency virus infected models [19]. Dopamine has also been found to induce the expression of HIV proteins in chronically HIV-infected T-lymphoblasts [20]. Our patient did not suffer any worsening of HIV and had steady improvement with levodopa and HAART for HIV-induced parkinsonism.

## Conclusions

Our case supports the safe and effective utilization of levodopa in conjunction with HAART in patients with HIV-induced parkinsonism. Further prospective studies with larger number of patients are likely to provide more information on this disorder.
